# Impact of Impedance Levels on Recording Quality in Flexible Neural Probes

**DOI:** 10.3390/s24072300

**Published:** 2024-04-04

**Authors:** Juyeon Han, Jungsik Choi, Hyeonyeong Jeong, Daerl Park, Eunji Cheong, Jaesuk Sung, Heon-Jin Choi

**Affiliations:** 1 Department of Materials Science and Engineering, Yonsei University, Seoul 03722, Republic of Korea; han_juyeon@yonsei.ac.kr (J.H.);; 2 Nformare Inc., Seodamun-gu, Seoul 03722, Republic of Korea; 3 Department of Biotechnology, Yonsei University, Seoul 03722, Republic of Korea

**Keywords:** neural probe, microelectrode, impedance, flexible polymer, coating

## Abstract

Flexible neural probes are attractive emerging technologies for brain recording because they can effectively record signals with minimal risk of brain damage. Reducing the electrode impedance of the probe before recording is a common practice of many researchers. However, studies investigating the impact of low impedance levels on high-quality recordings using flexible neural probes are lacking. In this study, we electrodeposited Pt onto a commercial flexible polyimide neural probe and investigated the relationship between the impedance level and the recording quality. The probe was inserted into the brains of anesthetized mice. The electrical signals of neurons in the brain, specifically the ventral posteromedial nucleus of the thalamus, were recorded at impedance levels of 50, 250, 500 and 1000 kΩ at 1 kHz. The study results demonstrated that as the impedance decreased, the quality of the signal recordings did not consistently improve. This suggests that extreme lowering of the impedance may not always be advantageous in the context of flexible neural probes.

## 1. Introduction

With the development of a general understanding of neuroscience, the demand for enhanced research instruments has increased. Neural probes offer an important neuro-technique applicable to basic and applied neuroscience, enabling real-time electrical recordings of neurons in living organisms [1]. Recently, several researchers have fabricated neural probes using various materials and techniques [1,2,3,4,5,6]. These innovations have yielded significant results in the understanding of brain functions and have improved the stability of recordings.

Many neural probes are fabricated using Si as the base material [2,3,4,6]. However, the rigidity and inherent fragility of Si pose challenges for the use of these probes in live animals, particularly for long-term implantation in active subjects [7,8]. Recent research has explored the use of flexible polymers such as polydimethylsiloxane (PDMS), parylene and polyimide as the base materials for neural probes [5,9,10,11,12]. These efforts enable us to maintain probe stability while minimizing damage to the brain tissue [11,13]. In addition to increasing in vivo stability through research on the base materials of neural probes, research aimed at obtaining high-quality signals is ongoing. Previous studies have shown that lowering the impedance of the electrodes can reduce noise and enhance their ability to detect spikes [14,15,16,17]. Consequently, recent studies have reduced electrode impedance using materials such as Au, Pt and poly (3,4-ethylenedioxythiophene) (PEDOT) to minimize noise in the fabrication of neural probes, thereby successfully conducting signal detection [5,6,18,19]. However, certain studies have presented the skeptical view that electrode impedance does not significantly affect spike detection [20].

Recently, a surge has been observed in research utilizing commercial neural probes [12,21,22,23], driven by the increasing interest in neuroscience across various fields. It has been a common practice in previous studies to coat electrodes of commercial neural probes before recording to reduce impedance and increase the quality of the recordings [24,25]. However, the extent to which one should reduce impedance and whether lowering impedance actually enhances recording quality in vivo remains unclear. Although one study investigated the relationship between impedance and recording quality using Si neural probes [24], studies on flexible neural probes that specifically investigate the impact of low impedance levels on high-quality recordings are lacking. Understanding the relationship between impedance and recording quality is crucial when employing flexible neural probes, as it could significantly enhance the accuracy of neural recordings, facilitating the development of more sophisticated and precise applications in neuroscience. Therefore, in this study, we employed a flexible neural probe to investigate the relationship between low impedance and recording quality.

## 2. Materials and Methods

### 2.1. Pt Electrodeposition and Measurement of Impedance of the Neural Probe

**Figure 1:**
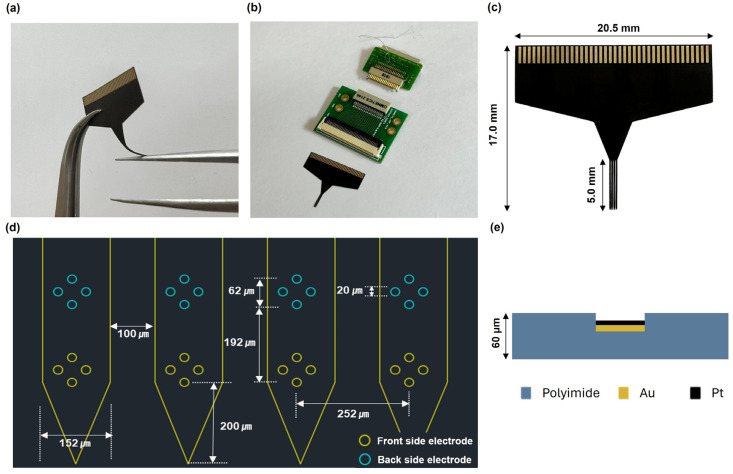
Design of the neural probe and a schematic of Pt deposition on Au electrode. (**a**–**c**) Picture of the device; (**d**) schematic of the tip of the probe; (**e**) schematic of the deposited Pt electrode.

**Figure A1:**
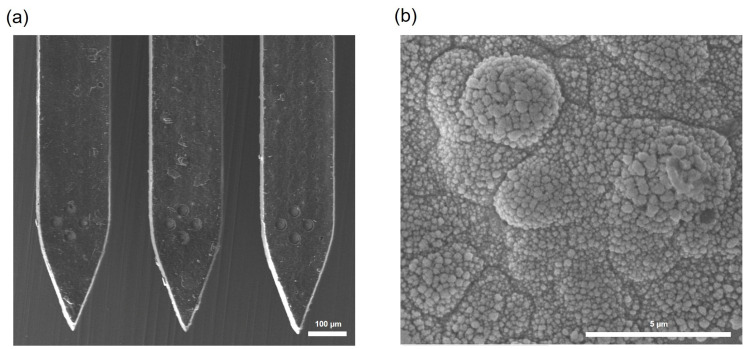
Scanning electron microscopy (SEM) image of the probe after Pt electrodeposition. (**a**) SEM image of shanks of the neural probe; (**b**) SEM image of the electrodes.

A commercial flexible polyimide neural probe (N32-1-B, Nformare, Seoul, Republic of Korea) was used. The probe type was a tetrode, and electrodes were placed on both sides of the shank (Figure 1a–c). The length, width and thickness of each shank of the neural probe were approximately 5 mm, 152 μm and 60 μm, respectively. The probe included four shanks; each shank had eight electrode sites of 20 μm diameter (area of 314 μm^2^), separated by 62 μm from center to center. The distance from the distant electrode to the edge of the shank was 200 μm, and the gap between each shank was 100 μm. Pt nanoparticles were deposited via electrodeposition to coat the Au electrode of the probe and control the impedance level of the probe (Figure 1d,e and Figure A1).

**Figure A2:**
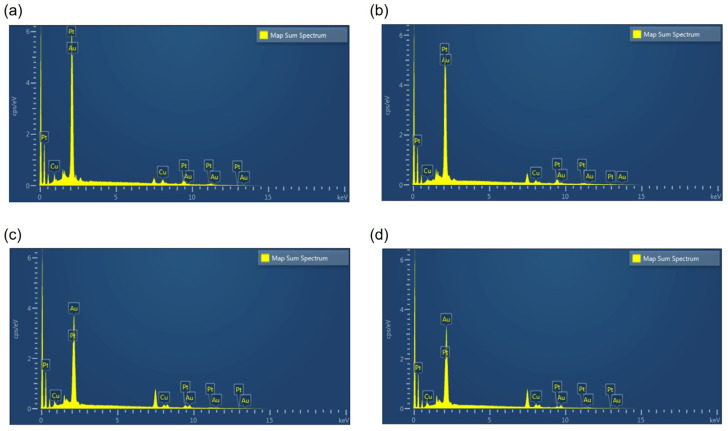
Energy-dispersive X-ray spectroscopy (EDS) data of the electrode. (**a**) 50 kΩ probe; (**b**) 250 kΩ probe; (**c**) 500 kΩ probe; (**d**) 1000 kΩ probe.

Electrodeposition and chronopotentiometry were performed using a potentiostat (VSP-300, BioLogic, Seyssinet-Pariset, France). Pt electrodeposition was performed in a three-electrode configuration using a solution of H_2_Cl_6_Pt (platinum black plating solution, Neuralynx, Bozeman, MT, USA). The working and counter electrodes were Au and Pt, respectively. A saturated Ag/AgCl electrode was used as the reference electrode. For neural recording applications, the optimal frequency is approximately 1 kHz [26]. This is because it corresponds to the typical duration of a neural spike that lasts for approximately 1 ms. Previous studies have reduced the impedance to 50 kΩ–1 MΩ as a conventional step to reduce the noise [5,6,18,19]. Thus, our study sought to explore whether electrode impedance affected spike detection by reducing the impedance within the 50 kΩ–1 MΩ range. We fabricated probes with impedance levels of 50, 250, 500 and 1000 kΩ at 1 kHz (pulse amplitude, 5–80 nA; pulse width, 5 s; recurrence period, 10 s; total time, 40 s). Electrochemical impedance spectroscopy (EIS) was performed to evaluate complex impedance before and after Pt electrodeposition. EIS was performed using a potentiostat. The tip of the probe was dipped in phosphate-buffered saline (PBS, Thermo Fisher Scientific, Seoul, Republic of Korea) solution. These measurements were performed with an AC potential of 10 mV applied across a frequency range of 1–5000 Hz. The composition of the electrode with respect to the impedance level was assessed using an energy-dispersive spectrometer (EDS, Octane Plus, Pleasanton, CA, USA) (Figure A2).

### 2.2. In Vivo Electrophysiological Recordings and Analysis

We conducted in vivo electrophysiological recordings of the ventral posteromedial nucleus (VPM) of the thalamus in anesthetized mice. The animals were cared for and handled in strict accordance with the guidelines established by the Institutional Animal Care and Use Committee of Yonsei University in Seoul, Korea. These mice were placed in a controlled environment with a 12:12 h light–dark cycle (with lights on at 7:00 a.m.) and unrestricted access to food and water. The VPM, a region of the brain involved in the somatosensory pathway, responds to whisker stimulation. Therefore, before recording, we inserted the probe into the VPM, stimulated the whiskers of the mouse and confirmed the probe’s capability to detect neuronal signals. Adult female C57BL/6J mice (12–16 weeks old) were used in this study. The experiments were conducted with three mice at each impedance level for a total of 12 mice. Mice were anesthetized with an intraperitoneal injection of urethane (1500 mg/kg). Subsequently, their heads were firmly placed in a stereotaxic device, and an incision was made in the scalp. A burr hole in the skull (approximately 2 × 2 mm in size) was carefully created above the VPM, following the established stereotactic coordinates for mice (centered at coordinates A/P −1.8 mm and M/L −1.8 mm from bregma, according to a previous study [27]). The dura mater was then gently removed. A probe was attached to a micrometric stereotaxic arm and connected to a head stage (HS-32-MUX, Neuralynx, USA), a connector (Nformare, Republic of Korea), an adapter (ADPT-HS36-N2T-32, Neuralynx, USA) and a Lablynx recording system (Neuralynx, USA) for data acquisition. The probe was lowered into the burr hole using the stereotaxic arm until it reached a depth of D/V from −3.6 to −3.8 mm for the VPM recordings. A stainless wire was inserted into the cerebellum as a reference electrode.

**Figure 2:**
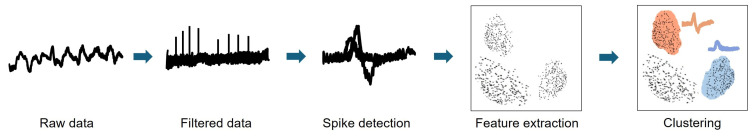
Process of signal processing and spike sorting.

In this study, we focused on the relationship between impedance levels and the ability to sort spikes from cells that could indicate the data quality. Through signal processing and spike sorting, we classified the cell clusters that represented the neuronal units. Figure 2 shows the signal processing process. The unprocessed electrode signals were recorded at a sampling rate of 30 kHz and saved for subsequent analyses using MATLAB 2019b (MathWorks, Natick, MA, USA). The recorded signals were processed through amplification and subsequent filtering within a bandpass range of 0.6–6 kHz that corresponds to the action potentials generated by neurons. Spike sorting was performed with reference to previous studies using the MClust 3.5 spike sorting software (A. D. Redish) for offline analysis [28,29,30]. The total energy of the spike waveform parameters was calculated for each channel, and the units were subsequently identified and isolated within the energy space. These clusters were then assessed and classified based on their potential representation of neuronal units using waveform analysis. Clusters that contained non-spike waveforms are colored black, whereas those containing spike waveforms are colored in other colors.

We investigated the number of clusters classified for each electrode. For each probe with impedance levels of 50, 250, 500 and 1000 KΩ at 1 kHz, we recorded the maximum number of classified clusters containing spike waveforms. After the analysis, the brain tissues were dissected from the mice to histologically verify the insertion track of the probe into the VPM. The brain tissues were isolated, preserved in formalin, embedded in paraffin blocks, and sliced into 5 μm thick sections. The slices were then mounted onto microscope slides and stained with 4′,6-diamidino-2-phenylindole (DAPI). After thorough rinsing, the slices were dehydrated and examined under an optical microscope.

## 3. Results

### 3.1. Pt Electrodeposition and Measurement of Impedance of the Neural Probe

**Figure 3:**
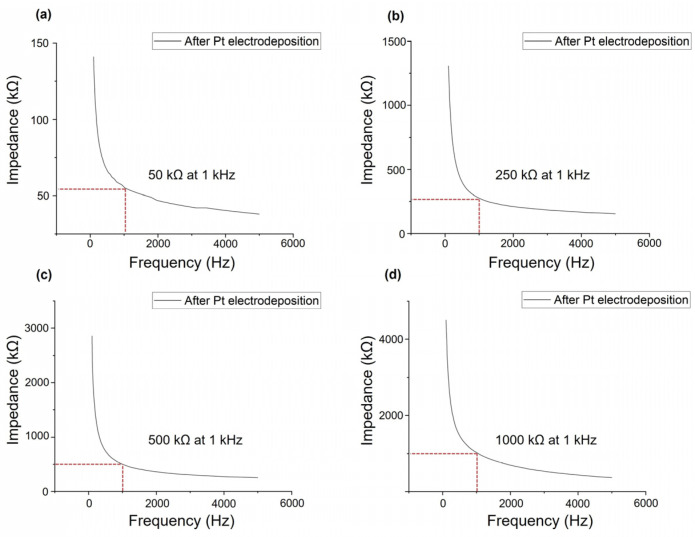
Representative impedance of electrode of electrodeposited neural probe at 1 kHz. (**a**) 50 kΩ probe; (**b**) 250 kΩ probe; (**c**) 500 kΩ probe; (**d**) 1000 kΩ probe.

The impedance level of each electrode was measured after the electrodeposition of Pt. The impedance level measured before Pt deposition was approximately 1.5 MΩ at 1 kHz. The mean impedance levels (SD) of the electrodes for each probe after Pt deposition were 50.4 (2.6) kΩ, 247.9 (7.2) kΩ, 504.4 (13.8) kΩ and 1027.4 (54.5) kΩ (Figure 3a–d). The deposition of Pt onto the Au electrode surface reduced the impedance, and the amount of deposited Pt could determine the impedance levels below 1 MΩ. The EDS analysis of each probe showed that the composition of Au decreased, whereas that of Pt increased compared with that before Pt deposition.

### 3.2. In Vivo Electrophysiological Recordings and Analysis

**Figure 4:**
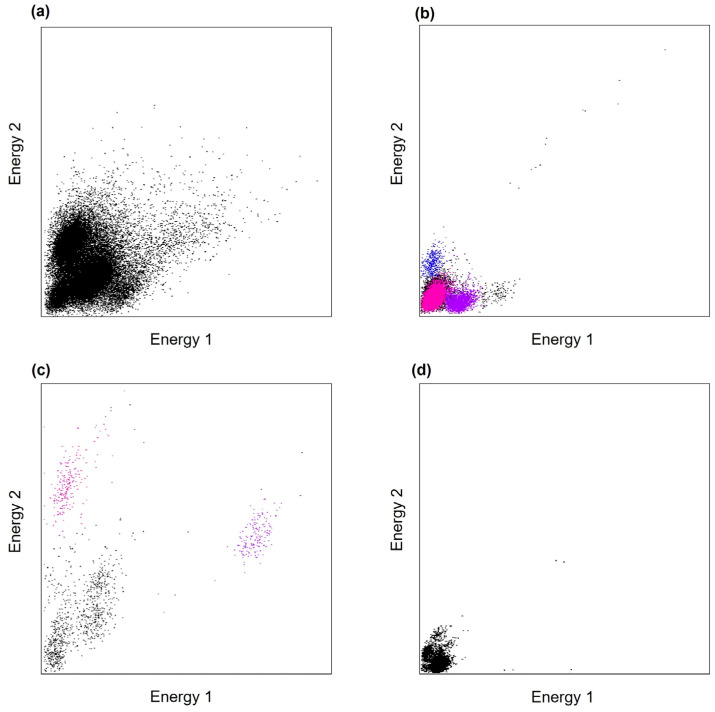
Representative electrophysiological recordings of cell clusters from anesthetized mice at different impedance levels. Each data point represents the energy, defined as the square root of the sum of the squared spike amplitudes. Clusters containing non-spike waveforms are colored in black, whereas clusters with spike waveforms are given various other colors. (**a**) 50 kΩ probe; (**b**) 250 kΩ probe; (**c**) 500 kΩ probe; (**d**) 1000 kΩ probe.

To evaluate the functionality of the probes with different impedance levels, we performed electrophysiological recordings of the VPM of anesthetized mice using three mice for each impedance level, for a total of 12 mice. Figure 4 shows the representative electrophysiological recordings from the electrode showing the maximum number of classified clusters in each probe with different impedance levels. The 50 kΩ probe recorded signals that could not be classified into clusters containing spike waveforms (Figure 4a). The 250 kΩ probe recorded signals that could be classified into cell clusters containing spike waveforms up to three (Figure 4b). Signals recorded from the 500 kΩ probe could be classified into cell clusters containing spike waveforms up to two (Figure 4c). Finally, the signals recorded from the 1000 kΩ probe could not be classified as cell clusters containing spike waveforms (Figure 4d).

**Figure 5:**
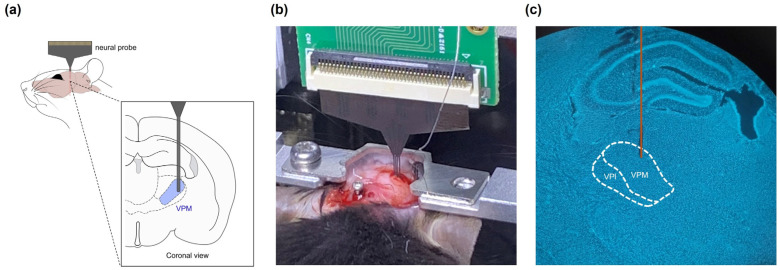
Schematic of the implanted probe and histologic confirmation of the insertion site. (**a**) Schematic of the implanted neural probe; (**b**) picture of the implanted neural probe; (**c**) neural probe insertion track (orange) in the VPM.

In this study, a polyimide-based flexible neural probe was selected. Polyimides are highly favorable materials because of their flexibility, stability and biocompatibility [9]. The probe exhibited significant flexibility, resulting in no mechanical failure during brain insertions. However, the high flexibility of this material can cause bending, thereby preventing it from reaching the desired location during insertion into the brain. Therefore, after the analysis, we histologically confirmed that the probe was successfully inserted into the VPM (Figure 5a–c).

## 4. Discussion

In this study, we investigated the relationship between the impedance level and the recording quality using a commercial flexible polyimide neural probe. The attenuation of neural signals owing to noise during recording is influenced by electrode impedance [31]. Previous electrophysiological studies have presented diverse perspectives on the influence of impedance on data quality [18,20,24,31,32,33,34]. The quality of the recordings depends on the ability to accurately detect the target signal from various signal sources. In the context of neural signal recordings, noise encompasses contributions that obscure the desired neuronal signals. Three main types of noise affect the signals detected by electrodes: intrinsic thermal noise inherent to the electrodes, background activity consisting of electrical signals from distant neurons that cannot be distinguished, and noise generated by recording amplifiers [31]. Among these, thermal noise is the primary source of noise that is significantly influenced by the impedance of the electrode, suggesting that adjusting the impedance of the probe electrode can modulate this type of noise [20]. Thermal noise is expressed using the following equation:

v^2^ = 4k_B_TZ, (1)

where v represents the noise amplitude (in units of volts per √Hz), k_B_ represents the Boltzmann constant, T denotes the temperature and Z denotes the impedance. As implied by the equation, an elevated impedance can increase the noise. Thus, a high impedance could lead to a more pronounced reduction in the signal detection quality. Consistent with previous research, we observed that high impedances (1000 kΩ) could detrimentally affect the signal quality. However, our results did not show consistent improvement in the ability to perform spike sorting through clustering as the impedance decreased from 1000 kΩ to 50 kΩ. As the impedance decreased from 1000 kΩ to 250 kΩ, spike sorting through clustering performed better. However, the performance at 50 kΩ was not better than that at 250 kΩ. A previous study conducted on the cortex and hippocampus of anesthetized rodents using commercial Si neural probes indicated that an impedance exceeding 2000 kΩ could lead to a decline in data quality [24]. However, the study suggested that an impedance range from 100 kΩ to 2000 kΩ might not significantly affect the data quality or the ability for spike sorting. Therefore, the authors suggested that an extreme reduction in impedance is not a strict necessity. Similar to the results of the previous study, our study also emphasized that an extremely low impedance, such as 50 kΩ, may not always be beneficial for flexible neural probes. Furthermore, unlike flexible neural probes, probes with thin and long insulated electrode wires, such as single microwires or tetrodes, can exhibit significant shunt capacitances [35]. Consequently, lowering the impedance may be advantageous because it reduces the loss of signals through shunt pathways. However, Si neural probes or recent neural probes using flexible materials have a considerably lower shunt capacitance, making them efficient in detecting spikes; hence, an extremely low impedance may not be considered a strict necessity [36].

In vivo electrode impedance properties may differ from those in vitro. Previous studies focusing on fabricating flexible neural probes generally aimed at lowering the impedance as much as possible [9,11,13]. This was based on in vitro studies that indicated that reduced impedance enhanced the signal-to-noise ratio (SNR), enabling high-quality recordings [18,32]. Whereas in vitro studies have demonstrated that cellular and biomolecular interactions increase with a decrease in the impedance of the electrodes [33], in vivo studies have suggested a weak association between recording quality and low impedance [24,34]. Noise can be considerably more variable in vivo than in in vitro conditions [24]. Whereas the reduction in impedance resulted in a decrease in non-biological noise, specifically thermal noise, this decrease was largely overshadowed by the significantly greater biological noise when considering in vivo conditions [34]. Consequently, an extremely low impedance may not contribute to enhanced spike detection. Moreover, within the realm of in vivo neural signal recording, tissue characteristics may have an effect. For example, certain brain regions contain densely packed layers of cells [11,37]. These densely packed cells, which are different from those in in vitro conditions, may impede neural signal detection at lower impedance levels. In addition, in vivo conditions can lead to abiotic or biotic changes in the electrodes. The corrosion of electrodes, along with tissue encapsulation owing to immune responses to foreign bodies, can alter the equivalent circuit models [38]. Therefore, as our results suggest, decreased impedance may not consistently improve the quality of signal recordings in in vivo conditions.

In this study, Pt electrodeposition was implemented for electrode coating. The reasons for coating the electrodes extend beyond merely reducing the impedance to improve spike detection. The aim is also to enhance cell stability against the electrode. For example, conductive polymers, particularly PEDOT, are used for electrode coating to increase physical and chemical stability and facilitate better contact with tissue [39,40]. Similarly, the Pt used in our research contributes to electrode stability, and recently, probes fabricated using PEDOT:PSS-coated platinum (Pt-PEDOT:PSS) microelectrodes, combining conductive polymer and Pt, have emerged [10]. Given the advantages of flexible probes, which include increased stability and minimized damage to the brain tissue, we anticipate an increase in research using flexible probes in the future. Coating these probes with new biocompatible conductive materials that can enhance their stability in in vivo experiments could significantly contribute to long-term applications. We believe that analysis through the long-term application of these neural probes could potentially contribute to improving our understanding the functions of the brain and neurological disorders.

This study is meaningful because it implies that exerting considerable effort to extremely reduce impedance through coating may not necessarily have a substantial impact on data quality. However, this study has certain limitations. First, owing to the small sample size, its generalizability is likely insufficient. Further studies are required to validate these findings. Second, we did not examine brain regions other than the VPM. Results might differ for other areas. Therefore, further investigations across diverse brain regions are required. Finally, we analyzed the results using limited analyses and statistical methods. For future research, advanced statistical analyses must be employed or machine learning algorithms must be applied to lend more weight to the conclusions regarding the relationship between impedance levels and recording quality.

The brain encompasses a multitude of core structures that are pivotal in regulating movement and emotional responses. Therefore, research dedicated to the study of the brain is of paramount importance. Advances in neural probe technology have considerable potential for both neuroscience and public health, paving the way for revolutionary treatments of neurological conditions and enhancing our understanding of the human brain. However, the progress of these technologies concurrently introduces a spectrum of ethical, legal and social considerations (ELSI) that necessitate careful deliberation. When conducting future research involving human subjects, potential ethical implications must be considered, thereby necessitating more meticulous and precise investigations. This study is important for neuroscience because it helps us to better understand the impact of impedance on the quality of signal recording when using flexible neuronal probes. The obtained results may be useful for further research on the development of more effective technologies for recording brain signals. In this context, our study contributes to the development of neural probes capable of yielding high-quality data, thereby significantly benefiting future research.

## 5. Conclusions

In this study, we electrodeposited Pt onto a commercial flexible polyimide neural probe to lower the impedance and conducted an in vivo study to investigate the relationship between the impedance level and recording quality. Our findings revealed that despite the decrease in impedance, no consistent improvement was observed in the classification of cell clusters. Moreover, our results indicate that excessively low impedance may not necessarily be advantageous for flexible neural probes. This study contributes to developing a better understanding of the relationship between impedance and data quality for future research on flexible neural probes.

## Data Availability

The data supporting the conclusions of this study will be made available by the authors on request.
